# Oral-genital human papillomavirus infection in Polish couples: frequent detection of HPV 42

**DOI:** 10.1186/s12879-018-3645-0

**Published:** 2019-02-06

**Authors:** Katarzyna Kiwerska, Agata Jozefiak, Janina Markowska, Witold Kedzia, Joanna Jackowska, Malgorzata Wierzbicka

**Affiliations:** 1 Institute of Human Genetics, Polish Academy of Sciences, Strzeszynska 32, 60-479 Poznan, Poland; 2 Department of Tumor Pathology, Greater Poland Cancer Center, Garbary 15, 61-866 Poznan, Poland; 3 Department of Preclinical Sciences and Infectious Diseases, Poznan University of Life Sciences, Wolynska 35, 60-637 Poznan, Poland; 4 Division of Gynecologic Oncology, Department of Oncology, Poznan University of Medical Sciences, Szamarzewskiego 82/84, 60-569 Poznan, Poland; 5 Department of Perinatology and Gynecology, Gynecology Clinic, Poznan University of Medical Sciences, Polna 33, 60-535 Poznan, Poland; 6 Department of Otolaryngology and Laryngeal Oncology, Poznan University of Medical Sciences, Przybyszewskiego 49, 60-355 Poznan, Poland

**Keywords:** Human papillomavirus, Cervical cancer, Oropharyngeal cancer, High-risk HPV, Low-risk HPV

## Abstract

**Background:**

Human papillomavirus (HPV) contributes to the development of cervical and oropharyngeal tumors. The increased incidence of HPV associated oropharyngeal tumors is lately being observed also in Polish population. The worldwide distribution of HPV varies and the studies rarely combine analysis of virus genotypes in both: genital and oropharyngeal locations. Therefore, in our study, we investigated HPV distribution in both anatomical sites of females with previous history of cervical cancer or with pre-cancerous lesion and their partners to establish the dominant types in Polish couples in genital and oropharyngeal areas, as they can be easily sexually transmitted.

**Methods:**

The study group consisted of 197 females and their partners. Each female had current or previous cervical pathology and HPV detected in gynecological swab with the use of Anyplex™ II HPV28 Detection system. This system is based on multiplexed real time PCR and enables detection of 19 high-risk and 9 low-risk HPVs.

**Results:**

Beside women, the virus was found in 114/197 of men in their foreskin swabs. Additionally, HPV was detected in oropharyngeal swabs of 39/197 female and 56/197 male participants. HPV 16/31/42/39/54 dominated in female and HPV 66/42/16/31/53 in male genital locations. The incidence of HPV in oropharynx was lower, top five genotypes included: HPV 6/39/42/35/16 in women compared to HPV 39/6/42/40/33 in men. HPV16 was the most frequently detected virus type, found in 70/197 examined cervical swabs. It was significantly more prevalent as single infection in females, previously treated for the cervical cancer (*p* = 0.035). Moreover, regular presence of low risk type 42 was noticeable in both sexes, in both kind of swabs. There was a trend observed towards its prevalence as single infectious agent in women with previous history of cervical cancer (*p* = 0.069).

**Conclusions:**

Our results showed the distribution of HPV genotypes in Polish couples, in which each woman is HPV positive, indicating a common infection of HPV 42, regardless of sex and anatomical site. These findings shed new light on HPV 42 significance, however they should be verified on a larger group of Polish participants, followed regularly in 6 months intervals, in oral as well as in genital areas.

**Electronic supplementary material:**

The online version of this article (10.1186/s12879-018-3645-0) contains supplementary material, which is available to authorized users.

## Background

In the last decades, there has been an increase in data concerning the incidence of HPV infections in humans. The most significant premise from these data is that HPV is the most important risk factor for the development of cervical cancer [1, 2]. According to GLOBOCAN reports, 15% of all tumors diagnosed in 2012 were causally related to the carcinogenic viral infections. Among all viral-associated cancers, the infection with HPV predominates (640,000 cases) and the vast majority of all HPV related tumors (530000) concerns cervical cancer (CC) developed by females [3, 4]. Beside other, rare, ano-genital locations (vulva, anus, vagina and penis), HPV may also inhabit the throat, leading to the development of oropharyngeal cancer (OPC) [2–4]. There are several factors that may facilitate this transmission, like early sexual initiation, the large number of sexual partners during the lifetime or the increased frequency of oro-genital intercourse. More than 90% of HPV infections are transient, being cleared by active immune response within 8 months [5, 6]. On the contrary, for the development of high-grade cervical intraepithelial neoplasia (CIN) and cervical cancer, viral persistence is essential but it may take more than two decades before the development of the abnormal lesions is observed [7].

HPV is small (50–55 nm in diameter) double stranded virus that exhibits tropism to epithelial cells (skin and mucosa). The virus genome length is around 8 kb and is divided into three regions: E (Early), L (Late) and LCR (Long Control Region). In general, HPVs constitute the heterogeneous group of viruses. So far, more than 200 mucosal and cutaneous subtypes were identified [8]. They are classified typically as high risk types (HR) - responsible for neoplastic transformation and low risk types (LR) - causing the benign hyperplastic lesions. The panel of HR HPVs includes among others types: 16, 18, 26, 31, 33, 34, 35, 39, 45, 51, 52, 53, 56, 58, 59, 66, 68, 70, 73 and 82, while the panel of types: 6, 11, 40, 42, 43, 44, 54, 61, 72 and 81 is considered as LR HPVs [1, 8–10].

The worldwide distribution of given genotypes varies among different localizations [1, 5, 11]. This also translates into the frequency of cancers caused by the viruses, i.e. HPV 16 and 18 are responsible for around 71% of cervical cancers, which occur mostly in less developed countries and for approximately 85% of head and neck cancers, which prevail in more developed countries [4, 12]. In Poland, as a result of numerous screening programs and social campaigns, the number of cervical cancer have decreased, but parallel to the global phenomenon, we observe the increase of oropharyngeal cancers [13]. Although currently three types of vaccinations (2-, 4- and 9-valent) are approved to be used preventively for Polish teenage girls and boys, our population should be treated as unvaccinated, as their long-term effectiveness will be observed when the vaccinated persons reach the age of the greatest incidence of cancer. Moreover, this vaccination is voluntary and payable as it was not included into Polish national immunization program. This condition has an undeniable effect on the low overall number of the vaccinated people.

HPV genotyping is essential for preventing cervical cancer and may also be useful for simultaneous screening of HPV in the oropharynx, as tumors of this region of head and neck seems to be most frequently associated with infection of these viruses. Therefore, in this study, we took an attempt to track the distribution of HPV genotypes in the genital and oral swabs of females with current or previous cervical pathology (each with ascertained HPV genital infection) and their partners, to determine which HPV types dominate in Polish, unvaccinated population and to find whether this distribution is similar in couples in both anatomical sites. Such report will enrich the knowledge concerning the distribution of HPV genotypes in female patients suffering from genital pathology and their asymptomatic partners.

## Methods

### Study group

The examined group consisted of 197 Polish female participants and their matched partners. Women were qualified to the group based on two premises: current or previous pathology of the cervix (including cancer) and HPV detected in the smear taken during gynecological examination. If a woman fulfilled these criteria, then her partner was included automatically to the study group. Women were diagnosed during routine cervical cytology or in the framework of preventive screening programs. The examinations were performed by specialists (gynecologists and oncologists) in “Boramed” Medical Center in Warsaw and two Clinics of Poznan University of Medical Sciences: Department of Gynecology and Department of Gynecological Oncology in Poznan, between years 2014–2016. The brush smears were taken with the use of Cervex-Brush® (Rovers, Oss, Holand) from the endocervix, ectocervix or cervical transformation-zone from women with CIN I–II or from the vaginal vault in patients after hysterectomy, performed previously to treat cervical cancer. From each patient’s partner the swab from the foreskin was taken with the use of FLOQSwabs (Copan, Brescia, Italy). Ultimately, 71 women with previous history of cervical cancer were included, out of which 18 had cancer detected in the year of material collection and 53 had cancer detected in the past (24 - one year before sampling, 22 – from two to nine years earlier and 7 – more than 10 years earlier). All of them were successfully treated for cervical cancer and during the study they were free of disease, with no symptoms of either recurrence or other malignancy. The remaining, 126 women had CIN I or CIN II detected (Additional file 1: Table S1).

In parallel, from each woman and man, the oropharyngeal swabs from tonsillar or tonsillar-lingual region were obtained with the use of FLOQSwabs. Oropharyngeal HPV positive patients were consulted at the Department of Otolaryngology, Head and Neck Surgery, Poznan University of Medical Sciences, Poznan, Poland. None of them presented any suspicious lesions in this anatomical site at the moment of the examination.

All samples were collected on ThinPrep liquid medium (Hologic Inc., Marlborough, USA). Each sample was designated for HPV genotyping with the same method. The experiments were performed in accordance with relevant guidelines and regulations, approved by the Ethics Review Board of Poznan University of Medical Sciences (decisions no. 21/13 and 75/15) and the written, informed consent was obtained from all participants.

### DNA isolation & HPV genotyping

DNA was isolated from 1 ml of hypercellular suspension containing gynecological or laryngological smear collected on ThinPrep liquid medium. The suspension was first centrifuged for 15 min at 13.000 rpm in order to separate cellular material from the brush or swab. Then, the supernatant was discarded and the pellet was suspended in 200 μl of 1 x PBS. Further, cell lysis, DNA precipitation, binding to the membrane and elution were performed according to procedure of NucleoSpin® Blood Kit (Macherey Nagel, Duren, Germany).

The HPV genotyping was performed with the use of Anyplex™ II HPV28 Detection system (Seegene, Seoul, Korea), which detects simultaneously 19 high-risk HPVs (16, 18, 26, 31, 33, 35, 39, 45, 51, 52, 53, 56, 58, 59, 66, 68, 69, 73, 82) and 9 low-risk HPVs (6, 11, 40, 42, 43, 44, 54, 61, 70) in a single specimen. This system, targeting L1 gene, is based on multiplex real-time PCR with high sensitivity and specificity due to the utilization of DPO™ (Dual-Priming Oligonucleotides) and TOCE™ (Tagging Oligonucleotide Cleavage and Extension) technologies. The reaction mixture of 20 μl included 1 x HPV28 primer mix A or B and 1 x Anyplex Master Mix. The real time PCR reaction was performed on a CFX96 Real-Time PCR thermocycler (Bio-Rad, Hercules, USA) with the following steps: 50 °C/4 min., 95 °C/15 min. and 50 cycles of 95 °C/30 s., 60 °C/60 s., 72 °C/30 s. Melting curve analysis was performed from 55 to 85 °C. The internal control - Beta-Globin (*HBB*) gene - was simultaneously amplified to ensure that the DNA amount is sufficient and that the PCR reaction is efficient. The positive (for each HPV type) and negative controls were included during each PCR run. The results were analyzed using the Seegene Viewer v. 2.0 program and were considered as valid, when internal control was present in each sample. A positive result (+++/++/+) indicated the presence of HPV DNA, while the negative result (−) indicated the absence of the HPV virus. The detection limit of the applied test is 50 copies of HPV per reaction.

### The statistical analysis

Chi - square exact test was used to evaluate the significance of given HPV/s in relation to women’s health status. It was performed with the application of free, online tool and the *p* value < 0.05 was considered as statistically significant.

## Results

### The qualitative and quantitative distribution of HPV types in different anatomical locations

The study group consisted of 197 Polish females with current or previous cervical pathology (cervical cancer treatment or cervical dysplasia) and HPV detected in the gynecological location as well as of their male partners. Each female patient was infected with at least one out of 28 types of the virus, detectable by the test (Additional file 1: Table S1). Additionally, 114/197 of their partners (57.9%) had at least one HPV type detected in the foreskin swab (Table 1). Concerning women health conditions, 71 out of 197 (36%) had been treated for the cervical cancer in the past. The remaining – 126 (64%), had cervical dysplasia detected (classified according to WHO criteria as cervical intraepithelial neoplasia: CIN I or CIN II; Table 1). Subsequent screening of HPV occurrence in the oropharyngeal swabs of each participant, revealed that the virus was present in the oropharynx of 39 females (19.8%) and 56 males (28.4%; Table 1). The stable relationship lasting for at least a year was declared by 112 couples (56.8%; Additional file 1: Table S1).

**Table 1:** The clinical data of participants and frequency of HPV in the collected swabs

Females (*n* = 197)		Males (*n* = 197)	
Age range			
20–64 years		24–65 years	
Age medium			
38.5 years		40.7 years	
HPV status in genital swabs			
HPV +	197/197 (100%)	HPV +	114/197 (57.9%)
HPV -	0/197 (0%)	HPV -	83/197 (42.1%)
HPV status in oropharyngeal swabs			
HPV +	39/197 (19.8%)	HPV +	56/197 (28.4%)
HPV -	158/197 (80.2%)	HPV -	141/197 (71.6%)
Cervix abnormalities in women			
Previously diagnosed cervical cancer-		71/197 (36%)	
CIN I or II -		126/197 (64%)	

**Fig. 1:**
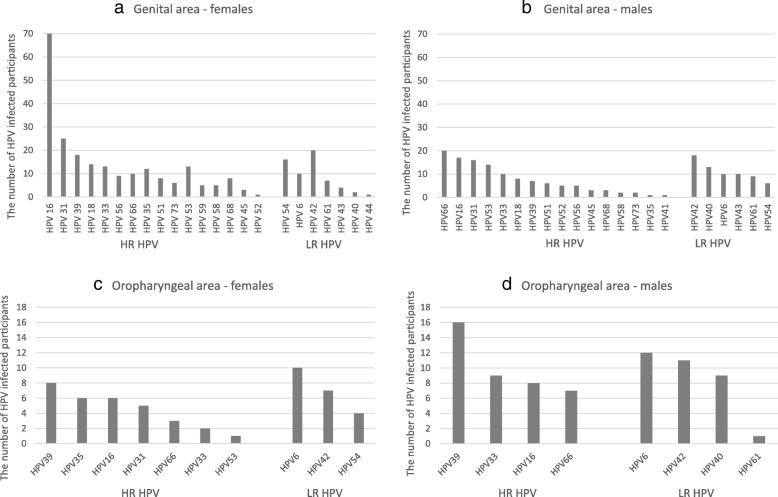
The prevalence of detected HR and LR HPV types, with reference to location (**a** and **b** - genital area; **c** and **d** - oropharyngeal area) and sex (**a** and **c** - distribution in females; **b** and **d** - distribution in males)

The distribution of HPV genotypes detected in particular swab groups in both sexes, according to their frequency is shown in Fig. 1 and Table 2.

**Table 2:** HPV types detected in clinical swabs according to their frequency

Females				Males			
Cervical swab		Oropharyngeal swab		Foreskin swab		Oropharyngeal swab	
HPV type	Number of positive samples	HPV type	Number of positive samples	HPV type	Number of positive samples	HPV type	Number of positive samples
HPV16	70	HPV6	10	HPV66	20	HPV39	16
HPV31	25	HPV39	8	HPV42	18	HPV6	12
HPV42	20	HPV42	7	HPV16	17	HPV42	11
HPV39	18	HPV35	6	HPV31	16	HPV40	9
HPV54	16	HPV16	6	HPV53	14	HPV33	9
HPV18	14	HPV31	5	HPV40	13	HPV16	8
HPV33	13	HPV54	4	HPV6	10	HPV66	7
HPV53	13	HPV66	3	HPV33	10	HPV61	1
HPV35	12	HPV33	2	HPV43	10		
HPV6	10	HPV53	1	HPV61	9		
HPV66	10			HPV18	8		
HPV56	9			HPV39	7		
HPV51	8			HPV51	6		
HPV68	8			HPV54	6		
HPV61	7			HPV52	5		
HPV73	6			HPV56	5		
HPV58	5			HPV45	3		
HPV59	5			HPV68	3		
HPV43	4			HPV58	2		
HPV45	3			HPV73	2		
HPV40	2			HPV35	1		
HPV44	1			HPV41	1		
HPV52	1						

Among these groups high risk as well as low risk types were observed. The top five, most frequently detected genotypes in genital smears included HPV 16/31/42/39/54 in women and HPV 66/42/16/31/53 in men (Table 2). The overall occurrence of HPV infection in oropharyngeal swabs was lower and the top five genotypes included: HPV 6/39/42/35/16 in women and HPV 39/6/42/40/33 in men (Table 2). The great predominance of HPV 16 in women’s gynecological swabs (70/197; 35.5%) was observed. The remaining types are rarely detected, although it should be noted that high risk types were present in each type of the swabs (Fig. 1). On the other hand, it was intriguing to observe the presence of one of the low risk viruses – HPV 42 among most frequent types in particular swabs (Fig. 1 and Table 2). In female participants, it was detected in 20/197 (10.2%) of gynecological swabs and in 7/39 (17.9%) of oropharyngeal swabs. In male participants it was detected in 18/114 (15.8%) of foreskin swabs and in 11/56 (19.6%) of oropharyngeal swabs.

Altogether, we have identified 114 couples (57.9%), in which both partners were infected in the genital area. In this group of couples, 33/114 pairs (28.9%) were infected with at least one concordant virus type. The stable relationship with one partner during the period of at least a year before the examination was declared by 22/33 pairs. In the remaining 81 couples (71.1%), the infection with distinct HPV types was determined (Table 3).

**Table 3:** The distribution of HPV infection in couples

The analyzed material		HPV infections in couples			Infection with one HPV type	Infection with ≥2 HPV types
		Infection of both partners	With concordant types^a^	With different types		
Genital swabs	Female	114/197 (57.9%)	33/114 (28.9%)	81/114 (71.1%)	131/197 (66.5%)	66/197 (33.5%)
	Male				62/114 (54.4%)	55/114 (45.6%)
Oropharyngeal swabs	Female	11/197 (5.6%)	9/11 (81.8%)	2/11 (18.2%)	30/39 (76.9%)	9/39 (23.1%)
	Male				47/56 (83.9%)	9/56 (16.1%)

^a^At least one concordant virus type was observed

The concordant types, detected more frequently (in > 10% of couples) included as follows: HPV 31, 16, 18, 66 and 42 (Table 4). In 11 couples (5.6%), the virus was detected in both: female and male oropharynx, in 9 out of these pairs both partners were infected by the same HPV type (6 or 33 or 39 or 42 or 66; Table 4). In the remaining two pairs, the infection included HPV 6 and 42 as well as 35 and 42. 6 out of 9 pairs with concordant viruses declared being in a stable relationship with one partner at least a year before the study.

**Table 4:** HPV types found in both partners of given couple

HPV type	Number of couples infected with at least one concordant HPV type according to the anatomical site of sampling	
	Genital area (*n* = 33)	Oropharyngeal area (*n* = 9)
6	1/33 (3%)	3/9 (33.3%)
16	6/33 (18.1%)	–
18	6/33 (18.1%)	–
31	8/33 (24.2%)	–
33	–	1/9 (11.1%)
39	–	2/9 (22.2%)
42	5/33 (15.2%)	1/9 (11.1%)
45	1/33 (3%)	–
56	1/33 (3%)	–
66	6/33 (18.1%)	2/9 (22.2%)

The infection with only one (any) HPV type predominated, however depending on the examined anatomical site and sex, the infection with more than two HPV types was also observed (Table 3).

### Analysis of HPV genotypes reveals high frequency of HPV42 in oro – genital location in females and males

HPV16 was the most frequently detected virus type, found in 70 out of 197 examined cervical swabs (35.5%). Within the group of HPV 16 positive females, 40 (57.1%) had been previously treated for the cervical cancer, while 30 (42.9%) presented low grade CINs at the time of study. The study shows that HPV 16 was significantly more prevalent in patients with the cervical tumor history in comparison to those, that had not developed tumor of this location (*p* = 0.002; Table 5). This finding was also statistically significant, when the infection was single, with only HPV 16 type (p = 0,035; Table 5).

**Table 5:** The frequency of infection with single or multiple HPV type in cervical pathology

A. Frequency of HPV 16 in relation to women’s health status				
Disease	Total number of patients (*n*=197)	Total number of patients with HPV 16 (*n*=70)	*p* value	
Previously diagnosed CC	71	40	0.002	
CIN I & II	126	30		
B. Most commonly detected HPVs as single or multiple infection in relation to women’s health status				
HPV 16 (*n*=70)	only HPV 16 (*n*=49)	HPV 16 + other types (*n*=21)	*p* value	
Previously diagnosed CC (*n*=40)	32	8	0.035	
CIN I & II (*n*=30)	17	13		
HPV 31 (*n*=25)	only HPV 31 (*n*=18)	HPV 31 + other types (*n*=7)	*p* value	*p* value with Yates’ correction*
Previously diagnosed CC (*n*=3)	1	2	0.112	0.366
CIN I & II (*n*=22)	17	5		
HPV 42 (*n*=20)	only HPV 42 (*n*=9)	HPV 42 + other types (*n*=11)	*p* value	*p* value with Yates’ correction
Previously diagnosed CC (*n*=15)	9	6	0.019	0.069
CIN I & II (*n*=5)	0	5		
HPV 39 (*n*=18)	only HPV 39 (*n*=3)	HPV 39 + other types (*n*=15)	*p* value	*p* value with Yates’ correction
Previously diagnosed CC (*n*=5)	0	5	0.239	0.638
CIN I & II (*n*=13)	3	10		
HPV 54 (*n*=16)	only HPV 54 (*n*=3)	HPV 54 + other types (*n*=13)	*p* value	*p* value with Yates’ correction
Previously diagnosed CC (*n*=3)	0	3	0.356	0.916
CIN I & II (*n*=13)	3	10		

*Yates’ correction was applied when at least one group consisted of less than 5 samples

Further, we have closely looked at the frequency of following four, most common HPV types in these patients. By order of the occurrence, HPV 31 was found in 25 women (12.7%), HPV 42 in 20 women (10.2%), HPV 39 in 18 women (9.1%) and HPV 54 in 16 women (8.1%; Table 2). Two types - HPV 31 and 39 - are high risk types, while the remaining two – HPV 42 and 54 – are low risk types. All but HPV 42 were more prevalent in patients without previous cervical cancer (pCC) history: HPV 31 was found in 22 CINs *vs* 3 pCCs, HPV 39 was found in 13 CINs *vs* 5 pCCs and HPV 54 was found in 13 CINs *vs* 3 pCCs (Table 5).

On the contrary, low risk type - HPV 42 - was observed more frequently in patients that had been treated for cervical cancer in the past (15 *vs* 5). It is noteworthy, that 9 out of 15 women with previously treated CC had HPV 42 detected as single infection. On the contrary, in none of the women with CINs (0/5) HPV 42 was detected as single one. Among 11 women who had HPV 42 and coinfection with other HPVs, high risk types dominated (Additional file 1: Table S1). This result showed only a trend towards statistical significance, as due to the small number of samples the applied Yates’ *p* value correction abolished it (*p* = 0.069; Table 5).

HPV 42 was also detected frequently in the oropharyngeal swabs, as third most common, both - in women (7/39; 17.9%) and in men (11/56; 16.6%). In women, HPV 6 and HPV 39 were detected more frequently (in 10 and in 8 women, respectively), while in men HPV 39 was most common (16/56; 25.6%), followed by HPV 6 (12/56; 21.4%).

## Discussion

HPV infection contributes to the cancer development in the mucosa of two distinctly distant anatomical sites: genital tract and upper aerodigestive tract. It is an indispensable factor of cervical cancer development but it may also affect other parts of female and male genitals. In the last decade, HPV detection rates have been increasing in the oropharyngeal cancers [3, 4]. This virus is sexually transmitted and its worldwide prevalence in both locations is common [2]. Contemporary, 60% of oropharyngeal tumors are associated with oral HPV infection - likely acquired from genital HPV infection during oral sexual behavior, leaving behind smoking - historically strongly associated risk factor [14].

The genotyping allows to establish the profile of particular HPVs in clinically involved tissues in different populations. It is often difficult to compare the results of HPV genotyping derived from different studies, as diverse methodology is used and various materials are analyzed. This may translate into false negative results [1, 11]. Additionally, the awareness, that during the lifetime man can be infected by HPV even several times and in most cases the infection is efficiently eliminated by the immune system but can somehow recur, is not conducive in obtaining unambiguous results [7]. Nevertheless, it is of great significance to widely analyze the distribution of particular HPVs in more local populations in order to create appropriate screening programs as well as to adjust novel vaccination combinations. Therefore, for our study we have selected only women with current or previous cervical pathology, in which we have further determined their current HPV genotype in the genital tract. To obtain complete information, regarding current virus types distribution throughout the body, additional samples from women and their partners have been taken. Almost all genotypes detectable by the applied test have been found. The most frequently detected genotypes were: HPV 16/31/42/39/54 in women genital swabs and HPV 66/42/16/31/53 in male foreskin as well as HPV 6/39/42/35/16 in female oropharynx and HPV 39/6/42/40/33 in male oropharyngeal area. The results of HPV genotyping in genital swabs generally fit into data observed by other researchers, but with slight difference – the low risk type, i.e. HPV 42 was present among most commonly detected types in each type of swab, in both sexes. Similar results were obtained by Fischer et al., who performed HPV testing on 615 Bavarian female patients with abnormal cytology – the three most commonly detected HPVs were the same: 16, 31 and 42. The remaining types in both studies were also detected but with different frequency [15]. Likely, compatible results were obtained by del Prete et al. who also found the prevalence of the same three HPV genotypes in women’s cervical/vaginal swabs, with HPV 42 as the most frequent [16]. The authors analyzed also male’s urethral swabs, indicating HPV 6, 42, 16 and 31 as most frequent, what is in line with our findings, with one exception - in our study HPV 66 but not HPV 6 was detected most frequently. Surprisingly, in both sexes HPV 18 did not prevail as it was expected [1, 3, 4]. The presence of HPV 42 among most common types detected in each sort of the swabs is striking and suggests that this type may be more prevalent in Polish population. The differences in type-specific prevalence of HPV in various worldwide locations is already shown by Munoz et al. [8]. Another interesting observation applies to HPV in relation to the health condition of the analyzed female cohort. Namely, 71/197 had a previous history of cervical cancer and in this group, HPV 16 and HPV 42 were detected more often than in women diagnosed with dysplasia. It is not surprising for HPV 16, which is the most prevalent high risk type worldwide, however for HPV 42 considered so far as low risk type this is rather intriguing [4, 8, 17]. The explanation could come from the study of Guimera et al., who proved association between single LR-HPV infection and anogenital carcinoma development. She found that HPV 42 and 70 are associated with squamous type of malignancy, showing diffuse p16 staining, typical for high-risk HPV-related tumors. At the same time, she indicated that infection with other low risk types, namely HPV 6/11 did not induce p16 overexpression. Moreover, the E7 sequence of HPV 42 and 70 contain pRB-binding motif and therefore both types could act in a similar way to HR HPV [18].

The genotypes in the individual couples overlapped partially: the concordant types were detected in 33/114 of couples in genital swabs and in 9/11 of couples in oropharyngeal swabs. In these groups, 22/33 and 6/9 couples, respectively, declared a stable relationship lasting at least a year. In the remaining couples we cannot be certain if the virus was transmitted between partners of a given pair. However, our result - 28.9% is concordant with the available data showing, that 13 to 63.2% of couples share the same HPV type in the genital area [19]. Nevertheless, our study shows that the concordant types were detected in couples more frequent in the oropharynx than in genitals, what might suggest a preferential inhabitation of this anatomical region by HPVs.

It should be highlighted, that neither male nor female participants had pathology in the oropharynx during laryngological examination. Similarly, none of the men reported any complaints from the penile. However, beside low risk types, the detected genotypes encompassed also high risk types, potentially causing cancer, therefore there is a constant need for these persons to perform regular check-ups. Especially in cases of oropharyngeal swabs, the positive HPV status is particularly disturbing in the light of recent results from Rosenthal et al., who found that normal, healthy controls lack HPV infection in their oral rinses [20]. The special attention should also be paid by men, while penile cancer, although rare, is a very aggressive malignancy and is associated with HPV infection in approximately 40% of cases [21].

## Conclusions

To conclude, our results shed lights on the distribution of HPV genotypes in Polish couples, in which female partner suffered from previously diagnosed cervical cancer or from cervical intraepithelial neoplasia grade I or II. We have pointed out the common infection with HPV 42 in women but also in their partners, in two anatomical sites: in the genital area as well as in the oropharynx. Importantly, HPV 42 was detected as the single infectious agent more often in females with previously diagnosed cervical cancer than in those with CIN I or II. Despite this, we cannot infer that HPV 42 should be reconsidered as high risk type in the general Polish population. Our findings should be verified on a larger group of Polish participants, including healthy controls, followed regularly in 6 months intervals, in oral as well as genital areas.

## Additional file

Additional file 1: **Table S1.** The characteristics of patients and their partners data. The file contains information concerning clinical data of the study participants, together with the results of HPV genotyping. (XLSX 29 kb)

## Abbreviations

CC: Cervical cancer
CIN: Cervical intraepithelial neoplasia
HPV: Human papillomavirus
HR: High risk
LCR: Long control region
LR: Low risk
OPC: Oropharyngeal cancer
